# The incidence of hypoglycaemia in Muslim patients with type 2 diabetes treated with sitagliptin or a sulphonylurea during Ramadan: a randomised trial

**DOI:** 10.1111/j.1742-1241.2011.02797.x

**Published:** 2011-11

**Authors:** S Al Sifri, A Basiounny, A Echtay, M Al Omari, I Harman-Boehm, G Kaddaha, K Al Tayeb, A S Mahfouz, A Al Elq, L Radican, C Özesen, H L Katzeff, B J Musser, S Suryawanshi, C J Girman, M J Davies, S S Engel, for the 2010 Ramadan Study Group

**Affiliations:** 1 Al Hada Military HospitalTaif, Saudi Arabia; 2 Diabetes InstituteCairo, Egypt; 3 Rafic Hariri University HospitalBeirut, Lebanon; 4 Jordan University of Science and TechnologyIrbid, Jordan; 5 Soroka University Medical CenterBeer Sheva, Israel; 6 Rashid HospitalDubai, United Arab Emirates; 7 King Abdullah Medical City Al-Noor HospitalMakkah, Saudi Arabia; 8 Bugshan HospitalJeddah, Saudi Arabia; 9 University of DammamDammam, Saudi Arabia; 10 Merck Sharp & DohmeWhitehouse Station, NJ, USA

## Abstract

**Aims:**

To compare the incidence of symptomatic hypoglycaemia in fasting Muslim patients with type 2 diabetes treated with sitagliptin or a sulphonylurea during Ramadan.

**Methods:**

Patients with type 2 diabetes (age ≥ 18 years) who were treated with a stable dose of a sulphonylurea with or without metformin for at least 3 months prior to screening, who had an HbA_1c_ < 10% and who expressed their intention to daytime fast during Ramadan were eligible for this open-label study. Patients were randomised in a 1 : 1 ratio to either switch to sitagliptin 100 mg qd or to remain on their prestudy sulphonylurea. Patients completed daily diary cards to document information on hypoglycaemic symptoms and complications. The primary end-point was the overall incidence of symptomatic hypoglycaemia recorded during Ramadan.

**Results:**

Of the 1066 patients randomised, 1021 (*n* = 507 for sitagliptin and *n* = 514 for sulphonylurea) returned at least one completed diary card and were included in the analysis. The proportion of patients who recorded one or more symptomatic hypoglycaemic events during Ramadan was lower in the sitagliptin group (6.7%) compared with the sulphonylurea group (13.2%). The risk of symptomatic hypoglycaemia was significantly decreased with sitagliptin relative to sulphonylurea treatment (Mantel–Haenszel relative risk ratio [95% CI] = 0.51 [0.34, 0.75]; p < 0.001). There were no reported events that required medical assistance (i.e. visits to physician or emergency room or hospitalisations) or were considered severe (i.e. events that caused loss of consciousness, seizure, coma or physical injury) during Ramadan.

**Conclusions:**

In Muslim patients with type 2 diabetes who observed the fast during Ramadan, switching to a sitagliptin-based regimen decreased the risk of hypoglycaemia compared with remaining on a sulphonylurea-based regimen. The incidence of hypoglycaemia was lower with gliclazide relative to the other sulphonylurea agents and similar to that observed with sitagliptin.

**What's known**

- Muslims are required to abstain from food and drink from dawn until dusk during the Islamic holy month of Ramadan.
- Many patients with diabetes fast during Ramadan despite the potential fasting-related risks and complications.
- The act of fasting and treatment with certain antihyperglycaemic agents increase the risk of hypoglycaemia in patients with type 2 diabetes.

**What's new**

- Treatment with sitagliptin, a DPP-4 inhibitor, reduces the risk of hypoglycaemia compared with a sulphonylurea in patients with type 2 diabetes who fast during Ramadan.
- Overall, treatment with sitagliptin or a sulphonylurea is well tolerated in these patients.

## Introduction

Millions of Muslims fast from dawn until dusk during the annual Islamic holy month of Ramadan. During the fast, Muslims are required to abstain not only from food and drink, but also from receiving oral medications. The Koran does exempt sick people from fasting. As the act of fasting increases the risk of hypoglycaemia in patients with diabetes, in part related to potentially impaired counter-regulatory responses to low blood glucose (1), treatment guidelines recommend that most patients with diabetes should not fast during Ramadan (2,3). However, in an epidemiologic study, the majority (78.8%) of patients with type 2 diabetes fasted for at least 15 days during Ramadan, with a 7.5-fold increase in the incidence of severe hypoglycaemia observed relative to the preceding months (4). The incidence of hypoglycaemic events was overall low, but was probably underestimated in this study, because only events requiring hospitalisation were counted. As many diabetic patients will fast during Ramadan despite the potential fasting-related risks and complications, the American Diabetes Association (ADA) published recommendations for managing diabetes during Ramadan (2).

There is no consensus about the most appropriate oral antihyperglycaemic agent(s) for patients with type 2 diabetes to use during Ramadan, as there are limited numbers of clinical trials assessing the efficacy and safety of these agents during Ramadan. The type of antihyperglycaemic therapy used may influence the risk of hypoglycaemia during a fast, with a higher rate of hypoglycaemia expected with oral agents that enhance insulin secretion in a non-glucose-dependent manner (5). Metformin is recommended because of the low risk of hypoglycaemia associated with its use, but the dose schedule may need to be altered to accommodate changes in meal patterns during Ramadan (2). Many patients with type 2 diabetes require additional antihyperglycaemic treatments to manage their disease (6). Sulphonylureas are typically recommended in combination with metformin because of broad clinical experience and lower cost (5). The ADA recommends caution when using sulphonylureas during Ramadan (2); sulphonylureas are associated with an increased risk of hypoglycaemia, but the risk varies across agents within this class (7,8). In a recent five-country observational study, the overall incidence of symptomatic hypoglycaemia was 20% during Ramadan in sulphonylurea-treated Muslims with type 2 diabetes, with a range of 14–26% with the different sulphonylurea agents (9). In small clinical trials, similar improvement in glycaemic control and a greater incidence of hypoglycaemia were observed with gliclazide relative to vildagliptin (10,11) and with glibenclamide relative to repaglinide (12) during Ramadan. Given their widespread use and potential for adverse effects, there is a need for additional clinical studies evaluating the effects of sulphonylurea compared with alternative treatment options in patients who fast during Ramadan.

Sitagliptin, a dipeptidyl peptidase-4 (DPP-4) inhibitor, has been shown to be effective and well tolerated with a low incidence of hypoglycaemia in clinical trials up to 2 years in duration (13–20). When added to ongoing metformin monotherapy, the addition of sitagliptin was shown to reduce the incidence of symptomatic hypoglycaemia, three to sixfold, compared with the addition of a sulphonylurea in patients with type 2 diabetes (18–20). Given the low risk of hypoglycaemia demonstrated in previous sitagliptin trials in non-fasting patients with type 2 diabetes, it was of interest to evaluate the incidence of hypoglycaemia with sitagliptin during Ramadan fasting. The present study was therefore undertaken to assess the incidence of hypoglycaemia with sitagliptin compared with sulphonylurea therapy (with or without metformin) in Muslim patients with type 2 diabetes who elected to fast during Ramadan.

## Methods

### Patients and study design

Eligible patients were Muslims with type 2 diabetes (age ≥ 18 years) who were treated with a stable dose of sulphonylurea [glimepiride, gliclazide (immediate or modified release) or glibenclamide (glyburide)] with or without metformin for at least the last 3 months prior to enrolment in the study, and had an HbA_1c_≤ 10% at the screening visit. In addition, patients expressed their intention to fast during Ramadan after receiving medical counselling regarding the risks of fasting and provided written informed consent. The inclusion and exclusion criteria for all patients were subject to verification by a clinical research associate during a routine site visit. Patients were excluded if they were treated with antihyperglycaemic agents other than a sulphonylurea with or without metformin, had a history of severe hypoglycaemia or had contraindications to treatment with DPP-4 inhibitors. Patients were recruited from clinical centres in Egypt, Israel, Jordan, Lebanon, Saudi Arabia and the United Arab Emirates (UAE). The study was designed in accordance with the principles stated in the Declaration of Helsinki. The protocol was reviewed and approved by the appropriate local authorities, as required, and by the institutional review board or ethical review committee for each participating clinical centre. The study was conducted from 17 June 2010 to 19 November 2010.

In this open-label study, eligible patients were randomised in a 1 : 1 ratio to either switch to sitagliptin 100 mg qd or to remain on their prestudy sulphonylurea (with or without metformin). For allocation to treatment group, each site was provided with a randomisation schedule. In countries where sitagliptin is indicated both as monotherapy and coadministered with metformin, randomisation was stratified by treatment regimen (monotherapy and coadministration). Following randomisation, patients and investigators were not blinded to treatment, and the study proceeded under real-life conditions without any additional protocol-mandated intervention. Physicians followed their patients as per usual clinical practice and were able to change drug and/or dose if needed to optimally manage their patients once Ramadan began.

At the screening visit (at least 5 weeks prior to the start of Ramadan), the following information was collected: age, gender, history of diabetes and related complications, weight, height, blood pressure and medication use. Blood samples were collected to measure HbA_1c_, fasting blood glucose, total cholesterol, low density lipoprotein cholesterol, high density lipoprotein cholesterol, triglycerides and serum creatinine.

For Ramadan, patients were provided with daily diary cards to record hypoglycaemic symptoms and complications, need for assistance due to symptoms of hypoglycaemia, time from consuming their last meal and time from taking their last medication dose to the start of the symptoms of hypoglycaemia and whether the fast was broken between dawn and dusk. Eating and drinking after a hypoglycaemic event was not considered a breaking-the-fast event in this study. If patients experienced symptoms of hypoglycaemia, they were instructed to perform fingerstick glucose measurements and to record the glucose results, as well as, the symptom(s) on their diary card. A diary card was to be completed by the patients on a daily basis throughout Ramadan, regardless of the presence of symptoms. If a patient experienced multiple symptomatic hypoglycaemic episodes on the same day, patients were to fill out diary cards for each episode. In addition, a preprandial blood glucose measurement was to be obtained prior to the evening meal three times per week on special colour coded diary cards. At the follow-up visit at the end of Ramadan (i.e. study end), additional information was collected including confirmation of observance of the fast during Ramadan and changes in diabetes medication dose and dose timing during Ramadan. Safety and tolerability were assessed by reviewing reported adverse events during the study. All adverse events were rated by the study site investigators for intensity (mild, moderate or severe) and relationship to study drug. Patients were also contacted by phone 2 weeks after Ramadan to assess the occurrence of any serious adverse events since study end.

### Outcome variables

The primary end-point was overall incidence of symptomatic hypoglycaemia recorded during Ramadan. Symptomatic events of hypoglycaemia included any event associated with clinical symptoms such as faintness, dizziness, headache, confusion, anxiety, sweating, tremor, palpitations, nausea, pallor and behavioural changes. The secondary end-point was the incidence of symptomatic or asymptomatic hypoglycaemic events [no reported symptoms, but a recorded blood glucose ≤ 70 mg/dl (3.9 mmol/l)]. Hypoglycaemic events were further categorised as: symptomatic events confirmed with a corresponding blood glucose value ≤ 70 mg/dl (3.9 mmol/l) or < 50 mg/dl (2.8 mmol/l), and severe hypoglycaemia was defined as events that caused loss of consciousness, seizure, coma or physical injury. In addition, hypoglycaemic events requiring assistance, either non-medical (e.g. family member or friend) or medical (e.g. visits to doctor's office or emergency room or hospitalisation), were also assessed.

### Statistics

Baseline demographics and clinical characteristics were summarised by treatment group. The All Patients as Treated (APaT) population was used as the primary analysis population for this study. The APaT population consisted of all randomised patients who received at least one dose of study treatment and returned at least one completed diary card during Ramadan. In addition, all patients were analysed in the treatment groups to which they were randomised, unless they took incorrect study medication for the entire treatment period. For the patients who changed antihyperglycaemic therapy after randomisation, only the hypoglycaemic events that occurred prior to the change were included in the analyses. Missing or incomplete diary cards were considered missing data. A supportive analysis using the Per-Protocol population was also performed. The Per-Protocol population was a subset of the APaT population, and included only those patients who completed the study and returned at least 70% of their completed diary cards. The primary and secondary end-points were assessed using a stratified Mantel–Haenszel test for the relative risk, with concomitant use of metformin therapy as a stratification factor. The total number of hypoglycaemic events in each study arm and the types of episodes were also summarised. A p-value < 0.05 (two-sided) was considered statistically significant. Assuming an incidence of symptomatic hypoglycaemia of 10% in sulphonylurea-treated patients during Ramadan [based on the results of Aravind et al. (9)] and that sitagliptin will reduce the risk by 50%, 434 patients per arm were required (two-sided α = 0.05, with a power of 80%). All data analyses were performed using SAS (Version 9.1.3; Cary, NC).

## Results

**Figure 1:**
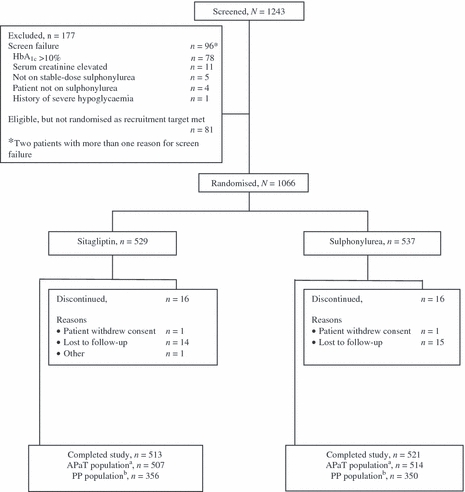
Patient disposition. ^a^The All Patients As Treated (APaT) population consisted of all randomised patients who received at least one dose of study treatment and returned at least one completed diary card during Ramadan. In addition, all patients were analysed in the treatment groups to which they were randomised, unless they took incorrect study medication for the entire treatment period. ^b^The Per-Protocol (PP) population was a subset of the APaT population, and included only those patients who completed the study and returned at least 70% of their diary cards completed

Investigators from 43 clinical sites in six countries screened 1243 patients, of whom 1066 were randomised to treatment (*n* = 529 for sitagliptin and *n* = 537 for sulphonylurea). Of the randomised patients, 1021 (95.8%) were included in the APaT population (Figure 1). Of the 45 patients excluded from the APaT population (*n* = 22 in the sitagliptin group and *n* = 23 in sulphonylurea group), 29 did not return or returned incomplete diary cards, 12 were lost to follow-up, two withdrew consent and two were withdrawn post randomisation for not meeting inclusion criteria (HbA_1c_ > 10% at screening visit and not on stable dose of sulphonylurea for at least 3 months prior to screening visit). For the APaT population, baseline characteristics were generally similar between treatment groups (Table 1). Overall, 51% of patients were men, mean age was 55 years and mean HbA_1c_ was 7.5% at baseline (Table 1). Patients had been on sulphonylurea treatment for a median of 4 years, 92% used sulphonylurea in combination with metformin and 15% self-reported experiencing a hypoglycaemic event in the 3 months prior to Ramadan (Table 2). For those who were randomised to remain on their prestudy sulphonylurea, 35% were treated with glibenclamide, 35% with glimepiride and 30% with gliclazide (Table 2).

**Table 1:** Baseline characteristics of randomised patients who returned at least one completed daily diary card during Ramadan

	Sitagliptin (*n* = 507)	Sulphonylurea (*n* = 514)
**Country**		
Egypt, *n* (%)	62 (12.2)	59 (11.5)
Israel, *n* (%)	117 (23.1)	122 (23.7)
Jordan, *n* (%)	45 (8.9)	42 (8.2)
Lebanon, *n* (%)	81 (16.0)	85 (16.5)
Saudi Arabia, *n* (%)	192 (37.9)	196 (38.1)
UAE, *n* (%)	10 (2.0)	10 (1.9)
**Patient characteristics**		
Age at baseline, years (range)	55 ± 11 (24, 94)	55 ± 10 (23, 87)
Gender male, *n* (%)	269 (53)	255 (50)
BMI, kg/m^2^	30.5 ± 5.7	30.5 ± 5.6
HbA_1c_, %	7.5 ± 1.3	7.6 ± 1.2
Fasting blood glucose, mg/dl	150 ± 52	153 ± 50
Duration of diabetes, years*	5.0	6.0
Systolic blood pressure, mmHg	130 ± 13	129 ± 14
Diastolic blood pressure, mmHg	78 ± 8	77 ± 8
Total cholesterol, mg/dl	179 ± 42	185 ± 43
LDL-cholesterol, mg/dl	106 ± 35	110 ± 36
HDL-cholesterol, mg/dl	42 ± 11	44 ± 18
Triglycerides, mg/dl*	148	150
Serum creatinine, mg/dl	0.7 ± 0.4	0.7 ± 0.4
**Diabetes- and cardiovascular-related complications and comorbidities**		
Neuropathy, *n* (%)	109 (22)	109 (21)
Retinopathy, *n* (%)	50 (10)	45 (9)
Nephropathy, *n* (%)	41 (8)	32 (6)
Coronary artery disease, *n* (%)	45 (9)	33 (7)
Peripheral arterial disease, *n* (%)	7 (1)	5 (1)
Cerebrovascular diseases, *n* (%)	8 (2)	3 (1)
Hypertension	214 (42)	221 (43)
Dyslipidaemia	316 (62)	317 (62)

* Median. Data are expressed as frequency, *n* (%) or mean ± SD unless otherwise indicated. To convert fasting blood glucose from mg/dl to mmol/l multiply by 0.0555. To convert cholesterol values from mg/dl to mmol/l multiply by 0.0259. To convert triglycerides from mg/dl to mmol/l multiply by 0.0113. To convert serum creatinine from mg/dl to μmol/l multiply by 88.4. UAE, United Arab Emirates; BMI, body mass index; MET, metformin.

**Table 2:** Sulphonylurea (SU) use prior to randomisation to treatment group

	Sitagliptin (*n* = 507)	Sulphonylurea (*n* = 514)
Glibenclamide	158 (31)	181 (35)
Glimepiride	189 (37)	178 (35)
Gliclazide	159 (32)	152 (30)
Monotherapy, *n* (%)	41 (8)	41 (8)
Dual: SU + MET, *n* (%)	465 (92)	471 (92)
Duration of SU therapy*, years	4.0	4.0
Experienced hypoglycaemia in 3 months prior to Ramadan	81 (16)	76 (15)

* Median. Data are expressed as frequency, *n* (%) unless otherwise indicated.

Based on returned diary cards, 93.7% of patients in the sitagliptin group and 89.7% of patients in the sulphonylurea group reported that they did not break the daytime fast (i.e. for reasons other than treating symptoms of hypoglycaemia) during Ramadan. The proportion of patients reporting a change in their diabetes medication dose or timing during Ramadan was 5.3% in the sitagliptin group and 6.6% in the sulphonylurea group. During Ramadan, eight patients in the sitagliptin group had their antihyperglycaemic therapy changed (seven to sulphonylurea and one to insulin). No patients in the sulphonylurea group changed their antihyperglycaemic therapy.

In the APaT population, the proportion of patients who recorded one or more symptomatic hypoglycaemic events during Ramadan was lower in the sitagliptin group (6.7%) compared with the sulphonylurea group (13.2%; Table 3). The risk of symptomatic hypoglycaemia was significantly decreased with sitagliptin relative to sulphonylurea treatment (Mantel–Haenszel relative risk ratio [95% CI] = 0.51 [0.34, 0.75]; p < 0.001). Among the patients randomised to remain on sulphonylurea treatment, the proportion of patients reporting symptomatic hypoglycaemic events was 19.7% (*n*/*n* = 36/183) in the glibenclamide, 12.4% (22/178) in the glimepiride and 6.6% (10/156) in the gliclazide subgroups. There was variability across countries in the proportion of patients reporting symptomatic hypoglycaemia (Table 3).

**Table 3:** Proportion of patients reporting symptomatic hypoglycaemia during Ramadan overall and by country

*n*/*N* (%)*	Sitagliptin	Sulphonylurea
Overall	34/507 (6.7)	68/514 (13.2)
Egypt	0/62 (0)	11/59 (18.6)
Israel	20/117 (17.1)	32/122 (26.2)
Jordan	3/45 (6.7)	3/42 (7.1)
Lebanon	8/81 (9.9)	19/85 (22.4)
Saudi Arabia	1/192 (0.5)	1/196 (0.5)
UAE	2/10 (20.0)	2/10 (20.0)

* No. of patients experiencing event/no. of patients overall or in each country by treatment (%).

Overall, a total of 323 symptomatic hypoglycaemic events were reported during Ramadan by patients in the APaT population, with 128 events in 34 patients in the sitagliptin group and 195 events in 68 patients in the sulphonylurea group. The number of patients reporting at least three symptomatic hypoglycaemic events during Ramadan was 22 in the sitagliptin group and 52 in the sulphonylurea group. The most commonly reported symptoms were headache, sweating, dizziness, hunger and tremor. The median time from the last meal to the onset of hypoglycaemic event was approximately 6 h.

Of the 1066 randomised patients, 706 (66.2%) patients met the Per-Protocol criteria (i.e. randomised patients who completed the study and returned at least 70% of their diary cards completed), with 356 patients in the sitagliptin group and 350 in the sulphonylurea group. In the Per-Protocol population, the proportion of patients who recorded one or more symptomatic hypoglycaemic events during Ramadan was 4.8% (*n* = 17) in the sitagliptin group and 14.3% (*n* = 50) in the sulphonylurea group (Mantel–Haenszel relative risk ratio [95% CI] = 0.33 [0.20, 0.57]; p < 0.001).

The proportion of patients with either symptomatic or asymptomatic hypoglycaemic events was 8.5% in the sitagliptin group and 17.9% in the sulphonylurea group (Table 4). The risk of symptomatic or asymptomatic hypoglycaemia was significantly decreased with sitagliptin relative to sulphonylurea treatment (Mantel–Haenszel relative risk ratio [95% CI] = 0.50 [0.36, 0.69]; p < 0.001). The proportion of patients with symptomatic hypoglycaemia confirmed with a corresponding blood glucose value ≤ 70 mg/dl (3.9 mmol/l) was 1.6% (*n* = 8) in the sitagliptin group and 4.3% (*n* = 22) in the sulphonylurea group. Two patients (0.4%) in the sitagliptin group and nine (1.8%) in the sulphonylurea group had a symptomatic hypoglycaemic event with a corresponding blood glucose value < 50 mg/dl (2.8 mmol/l). The incidence of hypoglycaemic events requiring non-medical assistance was low, with 0.2% of patients in the sitagliptin group and 0.8% in the sulphonylurea group. There were no reported events that required medical assistance (i.e. visits to physician or emergency room or hospitalisations) or were considered severe (i.e. events that caused loss of consciousness, seizure, coma or physical injury) during Ramadan (Table 4).

**Table 4:** Proportion of patients reporting hypoglycaemia during Ramadan by type* of event

	Sitagliptin (*n* = 507) no. (%) of patients experiencing event	Sulphonylurea (*n* = 514) no. (%) of patients experiencing event
Symptomatic or asymptomatic hypoglycaemic events	43 (8.5)	92 (17.9)
Severe hypoglycaemic events	0	0
Hypoglycaemic events requiring non-medical assistance	1 (0.2)	4 (0.8)
Hypoglycaemic events requiring medical assistance	0	0

* Types of hypoglycaemic event defined in Methods.

In addition to events of hypoglycaemia, 19 other adverse events were recorded during Ramadan. In the sitagliptin group, three patients reported a total of three adverse events: constipation, hyperglycaemia and vomiting. In the sulphonylurea group, nine patients reported a total of 16 adverse events: unstable angina, asthenia (*n* = 2 patients), cough, ischaemic stroke, acute pancreatitis, somnolence (*n* = 9) and urinary tract infection. No deaths were reported during Ramadan. Three of the aforementioned adverse events (ischaemic stroke, acute pancreatitis and urinary tract infection) in the sulphonylurea treatment group resulted in hospitalisation and thus were considered serious adverse events. No serious adverse events were reported for the sitagliptin group.

## Discussion

This large, prospective, randomised, multicentre study evaluated the incidence of symptomatic hypoglycaemia during Ramadan in Muslim patients with type 2 diabetes who remained on their prestudy sulphonylurea or switched to sitagliptin (with or without metformin). In this study, overall 92% reported that they did not break the daytime fast during Ramadan. The incidence of symptomatic hypoglycaemia in the APaT population was 6.7% with sitagliptin and 13.2% with sulphonylurea. Thus, the risk of symptomatic hypoglycaemia was decreased by nearly 50% with sitagliptin relative to sulphonylurea treatment. Moreover, in the Per-Protocol analysis, the risk of symptomatic hypoglycaemia was decreased by 67% with sitagliptin. These results were further supported by the lower incidence of symptomatic hypoglycaemia confirmed with a corresponding blood glucose value ≤ 70 mg/dl (3.9 mmol/l) with sitagliptin. There were no reports of severe hypoglycaemia, as defined in this study, and no reports of patients requiring hospitalisation or visits to their physicians due to a hypoglycaemic event. This is in contrast to previous reports of 0.5–2% of patients requiring hospitalisation for hypoglycaemia during Ramadan (4,9). The lower risk of hypoglycaemia with sitagliptin relative to sulphonylureas overall is consistent with the glucose-dependent actions of DPP-4 inhibitors relative to the non-glucose-dependent mechanism of action of potassium channel-based insulin secretagogues, such as sulphonylureas (5,21).

The difference in hypoglycaemia between sitagliptin and sulphonylurea was driven by the higher incidence of hypoglycaemia observed in the glibenclamide- and glimepiride-treated patients. The present findings are consistent with those from clinical trials not conducted during Ramadan that found a significantly lower incidence of hypoglycaemia with sitagliptin compared with glipizide or glimepiride (18–20). The incidence of hypoglycaemia was similar between sitagliptin and gliclazide in the present study. The lower incidence of hypoglycaemia observed in the gliclazide group relative to other sulphonylureas is consistent with results observed in other studies conducted during (9) and not during Ramadan (22). Thus, selection of sulphonylurea may impact the risk of hypoglycaemia during Ramadan.

Few clinical trials have compared the efficacy and safety of antihyperglycaemic agents in fasting patients with type 2 diabetes during Ramadan. In small observational studies (≤ 72 patients/study) from the UK, the incidence of hypoglycaemia was compared between Muslim patients with type 2 diabetes on vildagliptin, a DPP-4 inhibitor or gliclazide during Ramadan (10,11). In these trials, the proportion of patients with at least one hypoglycaemic episode was significantly lower with vildagliptin (0–8%) than with gliclazide (42–62%). Differences in study design (e.g. sample size, observational vs. randomised trial, definition of hypoglycaemia) may account for the higher incidence of hypoglycaemia reported with gliclazide relative to the incidence observed with sulphonylurea in the present study. In addition, in one of these prior studies (10), gliclazide was initiated at the beginning of Ramadan. It is possible that the higher incidence of hypoglycaemia was related to this fact, as hypoglycaemia rates may be higher during initiation of sulphonylurea therapy. Furthermore, the patients in that study were not randomly assigned to treatment with gliclazide, raising the possibility that a bias was introduced into the treatment assignments. Clearly, however, treatment with a DPP-4 inhibitor (sitagliptin or vildagliptin) reduces the incidence of hypoglycaemia relative to treatment with a sulphonylurea agent during Ramadan.

In another study using a treatment switch design, 235 Muslim patients with type 2 diabetes previously treated with a sulphonylurea were randomised to remain on a sulphonylurea (glibenclamide) or switch to repaglinide 6 weeks before Ramadan (12). Both treatments were titrated to optimal dose in the 6 weeks before Ramadan. During Ramadan, the incidence of symptomatic hypoglycaemia was 7% in the repaglinide group and 8% in the glibenclamide group. The incidence of confirmed symptomatic hypoglycaemia (events with a corresponding blood glucose value of < 2.8 mmol/l) was lower in the repaglinide group compared with the glibenclamide group (2% vs. 4%). In other smaller trials with various study designs, no differences were observed in the incidence of hypoglycaemic events between a sulphonylurea and active comparators (repaglinide, insulin glargine) (23,24).

The incidence of symptomatic hypoglycaemia varied by country in the present study. In particular, the proportion of patients reporting hypoglycaemia was unexpectedly low in patients from Saudi Arabia despite comprising nearly 40% of the randomised population. While there may be potential under-reporting of hypoglycaemia by patients from Saudi Arabia, review of the diary cards completed by all patients did not show any apparent study design-related factors that would affect the validity of the present findings. A similar pattern of country-specific variability was recently described in sulphonylurea-treated patients (9). In Aravind et al. (9), the proportion of patients reporting hypoglycaemia was lowest in patients from Saudi Arabia (10%) relative to the overall cohort (20%). Aravind et al. speculated that the differences across countries may be related to differences in patient characteristics, variations in physicians’ practices to modify the doses of antihyperglycaemic medications or the timing of administration to coincide with the fasting period of Ramadan or variations in dietary and lifestyle habits during Ramadan. Reasons for differences across countries were not assessed in the present study.

The following strengths and limitations of this study should be acknowledged. The study evaluated the incidence of hypoglycaemia using a randomised design with over 1000 Muslim patients from six different countries. Subjects were required to record hypoglycaemic symptoms (or no symptoms) daily during Ramadan rather than recall hypoglycaemic events at a final study visit. The primary end-point, symptomatic hypoglycaemic events, did not require a confirmatory blood glucose measurement, which may have overestimated hypoglycaemic events. However, the findings of symptomatic hypoglycaemia with a corresponding blood glucose ≤ 70 mg/dl [3.9 mmol/l] support the primary findings of a higher incidence of symptomatic hypoglycaemia with sulphonylurea compared with sitagliptin. Although 15% of patients self-reported experiencing a hypoglycaemic event on their sulphonylurea-based regimen in the 3 months prior to Ramadan, the incidence of hypoglycaemia was not formally assessed with sitagliptin or sulphonylurea before or after Ramadan. The effect of treatment on glycaemic control and body weight was not assessed during Ramadan in the present study. In previous clinical trials not conducted during Ramadan, the glucose-lowering efficacy of sitagliptin and a sulphonylurea (glipizide or glimepiride) was similar, whereas treatment with sitagliptin led to weight loss relative to weight gain with the sulphonylurea (18–20).

## Conclusions

In Muslim patients with type 2 diabetes who observed the fast during Ramadan, switching treatment to a sitagliptin-based regimen decreased the risk of hypoglycaemia compared with remaining on a sulphonylurea-based regimen. In this study, the incidence of hypoglycaemia was lower with gliclazide relative to the other sulphonylurea agents and similar to that observed with sitagliptin. Both treatment regimens were generally well tolerated during the month of Ramadan.

## Appendix

Ramadan 2010 study investigators by country: Egypt: Atef Basiounny, Hisham Elgayyar, Salah Ghazaly Harb, Khalifa Mahmoud; Israel: Yones Aburabia, Faiad Adawi, Halima Dabaja, Mahmud Daraushe, Shadi Hamoud, Ilana Harman-Boehm, Tony Hayek, Jarir Khuri, Abdallah Mashal, Dakuar Nakhly, Mohamad Otman, Mochmad Sabbah, Naim Shehadeh, Adnan Zaina, Agrabrya Zoadi, Taufik Zuabi; Jordan: Omar Abuhijleh, Mohammad Alzaheri, Nidal Khatib, Mousa Al Omari; Lebanon: Sobhi Dane, Akram Echtay, Youssef Hawly, Rafic Kanaan, Abdel Amir Khalifeh, Marwan Krayem, Hicham El-Nazer, Haytham Rahhal, Samar Saad; Saudi Arabia: Issa Aldhafiri, Mourad Elmourad, Abdulmohsen Al Elq, Ashraf Shaaban Mahfouz, Shahid Omar, Saud Al Sifiri, Khaled Al Tayeb; United Arab Emirates: Fatheya Al Awadhi, Ahmed Hassoun, Ghaida Kaddaha, Alaa El Din Khidr.
